# Communication Surrounding Estrangement: Stereotypes, Attitudes, and (Non)Accommodation Strategies

**DOI:** 10.3390/bs8100096

**Published:** 2018-10-20

**Authors:** Christine Rittenour, Stephen Kromka, Sara Pitts, Margaret Thorwart, Janelle Vickers, Kaitlyn Whyte

**Affiliations:** Department of Communication Studies, West Virginia University, Morgantown, WV 26506, USA; smk0023@mix.wvu.edu (S.K.); sep0014@mix.wvu.edu (S.P.); mat0039@mix.wvu.edu (M.T.); jpvickers@mix.wvu.edu (J.V.); kvwhyte@mix.wvu.edu (K.W.)

**Keywords:** stereotypes, estrangement, contact hypothesis, stereotype content model, parent‒child, communication accommodation theory

## Abstract

To address Americans’ general attitudes and behavioral intentions toward adult children who are estranged from their parents, the current study employed online survey data from 151 Americans recruited through Amazon MTurk. Their responses revealed negative stereotypes (e.g., childish, ungrateful) and positive stereotypes (e.g., independent, strong) of the adult child who is estranged, as well as negative assessments of the parent who is estranged. Generally, participants perceived the adult children as more competent than warm. Compared to other participants in this sample, those participants who were estrangers or estrangees themselves held more positive attitudes overall, including more positive perceptions of estranged children’s warmth and competence. In response to open-ended survey questions asking participants how they would communicate with someone they knew to be estranged, common responses were avoidance of family-related topics, (heightened) physical distance, and accommodation to the needs of the person who is estranged. Implications are discussed surrounding the lack of warmth associated with those experiencing estrangement.

## 1. Introduction

While it is both an expectation and standard that family members—particularly parents and children—will remain close [1], ongoing difficulties among family members can ultimately lead to estrangement. Supplementing literature on within-family communication surrounding estrangement, the current study addresses the macro discourse surrounding the phenomenon. Societal views are of great consequence to the groups they target, and are well captured through stereotypes—the oversimplified qualities and characteristics held about a targeted group [2]. As stereotypes are communicated *and* stem from communication, they are of great importance to our understanding of interactions with estranged individuals. The first goal of this study is to examine Americans’ stereotypes of adult children who are estranged from a parent. The second goal is to explore how intergroup contact (Intergroup Contact Hypothesis [3]) and self-identification correspond with Americans’ attitudes regarding estranged adult children. The third goal is to address the strategies people employ when interacting with someone known to be estranged (Communication Accommodation Theory [4]).

### 1.1. Estrangement

Parents and children are expected to have deep, meaningful, and caring communication exchanges that endure in good and bad times [1], but estranged family relationships—those that are dormant, distanced, or dissolved—are common, even between parents and their children [5]. While estrangement is an ongoing process in which one or both parties are actively communicating to adjust and renegotiate intimacy boundaries [6,7], *parental estrangement* is often the result of the child wanting to decrease their parent’s unwanted involvement [8]. These adult child estrangers report betrayal, parental indifference, and lack of support, inclusion, and acceptance among their reasons for distancing [9] with some relationships involving abuse [6,10,11]. Intensifying of the estrangement can occur in linear and non-linear trajectories, with the latter including a “cycle of reunification” whereby an adult child attempts to reconnect with a parent, discovers that the behaviors or opinions that caused the estrangement had not changed, and then chooses to remove him or herself again from the relationship [6,10]. Even when children feel confident in their decision to cease contact, they experience great pain [7]. As the estrangees, parents in such circumstances also see this as traumatic, and are sometimes shocked by the rejection by a family member, presuming this relationship would always be intact, even if strained [11]. Parents in this process can also make negative attributions for their estranged children, such as entitlement and lack of appreciation [9]. While less documented, it also occurs that parents are the estrangers and children the estrangees. In addition to negative experiences and/or the absence of positive experiences within the parent‒child dyad, the internal struggles surrounding this familial disconnection and reconnection might be influenced by external pressures. Adult children are likely influenced by pressures from social networks that encourage reconciliation and stress that family relationships should never be dissolved [10], and may internalize cultural expectations to reunite with their parent [6].

Even though estrangement is a phenomenon confined to the family system, it is strongly linked to judgment from outside of the family [12], with both the parent and child in the estrangement dyad fearing societal judgment. Recently, Agllias described how our idealized notions about the strength, safety, and stability of family relationships are what exacerbate the pain of the estrangement [7]. Estranged parents expressed a sense of social isolation, feeling more limited in the number and variety of topics available to discuss with others [13,14]. Many estrangers and estrangees do not mention their estrangement to others out of a fear of judgment by their social network, presuming that this placed an inappropriate burden that prohibited its sharing [15]. This judgment might be particularly strong for mothers [12], who are the presumed primary kinkeepers of the family [16] and prone to being stereotyped, even within intact traditional family systems [17]. While men are considered less apt at parenting [18] , their stereotypes become more positive when they are identified as fathers rather than “just” men [18], but women’s heightened expectations for sound parenting are what place them at risk for heightened speculation. Mothers who are estranged report that society judges them in their parent role and for the perceived bad behavior of their estranged adult children [13]. A daughter in Agllias’s [7] research references stigmatizing comments from those claiming she should reconnect (simply) because “it’s your *mother*.” These emphasized gendered expectations serve to further stigmatize these hurting families. The current study targets the gaze placed upon estranged children with the intention of informing efforts to reduce the negative reaction that estranged adult children perceive and receive.

### 1.2. Categorizations and Stereotypes

Estranged adult children’s fears of judgment by their social network [15] might be justified. Because there are expectations and reciprocal benefits to well-being for adult children having healthy relationships with parents [19,20], adult children who are estranged from a parent face additional hardship. To create social distance from those who are estranged (“that won’t happen to me”), onlookers might place an added level of civil scrutiny on estranged children.

According to the social identity theory of intergroup communication [21] and its successor, self-categorization theory [22], individuals strive to attain a positive identity through the groups in which they belong. This process is facilitated by making social comparisons between one’s ingroup and the (less positive) outgroups. This categorization of self and other promotes exaggerations of differences between groups, which lead to stereotyping and prejudicial attitudes toward outgroup members, all of which serve to constrain communication exchanges and are potentially harmful to the self-concept [22].

While family roles are not a typical context for intergroup research, probably because scholars and laypeople uphold Allport’s initial assumption that families comprise our deepest ingroup [3], Soliz and Rittenour describe how families are ripe with intergroup dynamics [23]. Structural and social distinctions within the family reveal how this micro context can mirror the macro-, and even global, intergroup dynamics of exclusion and hostile treatment [24,25]. In addition to those within the family harboring negative attitudes and assumptions about their “different” family members—an underlying mechanism of estrangement processes [14]—the overall structure of a family also cues stereotypes among perceivers [17]. Specific roles within the family have also been viewed from an intergroup perspective, with researchers demonstrating how society judges outgroups of stay-at-home and working mothers [26] as well as gay fathers [27]. As family is the site of heightened scrutiny, overgeneralization, distortion, and simplification, those family members severing the iconic parent‒child relationship are likely to be viewed negatively and stereotyped accordingly. Critical scholars assert that societal designations of those who are estranged as unnatural and dysfunctional only perpetuate the problem [28]. We anticipate this negativity will emerge in the stereotypes we aim to assess.

Lippmann [29] described stereotypes as “pictures in our heads” that overgeneralize a social group’s characteristics and behaviors. Humans are prone to cuing stereotypes quickly and acting in accordance with those stereotypes rather than basing their cognitive and communicative responses on the actual behaviors of individual outgroup members [2]. As such, stereotypes keep us from knowing each other as unique, complex individuals, which in turn prohibits people’s relational closeness and positivity. Identifying specific stereotypes held about a social group allows researchers to pinpoint the specific ways the target group (i.e., adult children who are estranged) is being “cognitively reduced” by outgroups.

We presume that family estrangement is somewhat of a taboo discussion topic, and we do not yet know the exact stereotypes that are socially created, shared, and replicated. Because estrangement goes against the notion of a traditional family structure’s presumed stability [30], we presume that stereotypes will reveal Americans’ criticisms of the behaviors they presume to surround estrangement. For instance, aforementioned trends lead us to assert that stereotypes will designate estranged family members as selfish, nontraditional, and at fault. But criticisms about poor family communication preceding the estrangement [13], could ignite compassion for a presumed innocent family member. In this case, we anticipate additional stereotypes of estranged children as being strong, courageous, or other more positive characteristics. Still, given the iconic nature of families and the parent‒child relationship, we anticipate that society will wish to distance themselves from these circumstances, leading to more negative than positive stereotypes overall. Therefore, we begin by positing the following research question and hypothesis:

**RQ1:**  What stereotypes do Americans hold of children who are estranged from their parents?

**Hypothesis 1** **(H1).** *Americans will report more negative than positive stereotypes of children estranged from a parent.*

Against our first research question and hypothesis’s broad interest in complete and specific stereotypes of people who are estranged, we target two stereotypes to reveal societal notions about specific target groups. The stereotype content model [31,32] posits these as the two universal dimensions of social cognition that comprise stereotypes. Warmth is the degree to which a stereotyped social group is appraised as acting in ways that are kind and in accordance with the other group’s goals. Competence is the degree to which that stereotyped social group is thought to be capable of enacting harmful and/or hurtful behaviors to the other group’s goals.

The warmth and competence that people perceive of estranged adult children has yet to be assessed, but we expect low scorings for both of these dimensions, particular regarding warmth. Family relationships are presumed to be everlasting, and so the absence of this relationship might trigger assumptions that the estranged child (and his/her parent) is possibly incompetent, and—even more likely—cold. Certainly, many of the aforementioned precursors to child-parent estrangement (i.e., abuse, poor parenting, and betrayal) suggest that estrangement is warranted, but stereotyping exists adjacently to facts [2]. Still, some communication surrounding estranged individuals might address the failed relationship and parents’ incompetencies, and this communication about the estranged relationship might suggest that the perceived competence of estranged adult children would not suffer so much as their perceived warmth (and perhaps warmth would be less low).

Given that our mere presumptions are not supported by any previous findings, we pose this research question in order to ultimately employ the stereotype content model’s utility of predicting the bias climate that this social group is likely to experience [33]:

**RQ2:**  What are Americans’ perceptions of estranged adult children’s warmth and competence?

### 1.3. Intergroup Contact and Improved Attitudes

We assert that research question two’s resultant warmth and competence designation will be different for participants with and without a personal history of estrangement. The contact hypothesis states that interaction between members of two outgroups will result in reduced prejudice and a less rigid reliance on stereotypes [3]. This interpersonal communication between individuals from different outgroups has consistently been shown to improve attitudes for not only the participating individuals, but also extending to both individuals’ respective social groups [34].

Although the current retrospective self-report study does not directly test Allport’s contact hypothesis, this highly heuristic theory leads to our prediction that either knowing someone who is estranged or self-identifying as having an estranged relationship will correspond with more positive perceptions of this communicative and relational process, while those who have not had such interactions will report less positive attitudes toward estrangers and estrangees. Thus, the following hypothesis is posited:

**Hypothesis 2** **(H2).** *Compared to Americans with no contact with estrangement, Americans with a personal history of estrangement (i.e., they know someone who is estranged, or they themselves are estranged from a parent) will perceive individuals who are estranged from a parent as more competent and warm.*

### 1.4. Exploring Communication Accommodations

As it addresses the many ways that individuals accommodate their communication with others as well as their motivations and consequences, communication accommodation theory (CAT) directs our final research question [35]. According to CAT, interactants (i.e., outgroup members) use convergent or divergent strategies when communicating with conversational partners. A convergent strategy is used when an interactant adapts to be more similar to a conversational partner. A divergent strategy is employed when an interactant accentuates the differences between themselves and a conversational partner [36]. These strategies lead to more similar or dissimilar means of communicating, with convergence generating greater feelings of liking and closeness.

In a recent meta-analysis, Soliz and Giles [35] designate CAT behaviors into these four general categories: accommodation, nonaccommodation, reluctant accommodation, and avoidant communication. Accommodation is defined as behaviors that one or both communicative partners use to be perceived as positive or conversationally appropriate with the other. Nonaccommodation is defined as behaviors in which communicative partners fail to adequately adjust their communication to fit the needs or desires of the other. Essentially, accommodation occurs when the communicator “hits the mark” and is appropriate to the unique, individualized needs of the recipient (i.e., the estranged adult child). Within the context of nonaccommodation, an individual might be underaccommodating (i.e., not adjusting their communication behaviors enough) or overaccommodating (i.e., adjusting their communication behaviors too much), with this overaccommodation often taking the form of patronizing behaviors [37]. Reluctant accommodation occurs when accommodation is grounded in obligation rather than as an effort for relational closeness. Avoidant communication involves ending the current interaction quickly and avoiding future interaction by constraining communication. Avoidant communication has been associated with unconscious negative stereotypes or previous destructive experiences with the perceived outgroup, and is expected to emerge within our study [38].

When individuals overestimate the differences between themselves and presumed outgroup members, they are inevitably going to think about these differences more than they should and communicate in accordance with that view [2]. If people draw from the hypothesized negative stereotypes when talking to someone who is estranged, they might appropriately accommodate by asking them about their desires to share their experiences. However, previous research suggests we often “miss the mark” by an unprompted (i.e., not being told “I don’t want to discuss my estrangement”) avoidance of the topic for fear of upsetting the individual, not knowing what to do in the conversation, or by getting upset with them. The accommodative and nonaccommodative behaviors people display toward estranged individuals are quite consequential, given that nonaccommodation is often dissatisfying at best [39], and can be just as disempowering as overaccommodation [40]. Thus, we propose a research question inquiring about these specific accommodative and non-accommodative behaviors directed toward people who are estranged from a parent.

**RQ3:**  What accommodative strategies will Americans report toward adult children estranged from a parent?

## 2. Method

### 2.1. Participants

Of the 214 participants, only one reported living outside of America and so was excluded from analyses for consistency. There were 63 who did not complete other demographic information. Among the 151, 62 (41.0%) reported living in a suburban area, 51 (33.8%) in an urban area, and 38 (25.2%) in a rural area. Seventeen participants reported being aged 18–24 (11.3%), 70 participants were 25–34 (46.4%), 31 participants were 35–44 (20.5%), 18 participants were 45–54 (11.9%), eight were 55–64 (5.3%), and seven were age 65 or older (4.6%). Sixty-three (29.4%) reported as male, 85 (39.7%) female, two (0.9%) nonbinary, and one (0.5%) female-to-male transgender. In regard to race/ethnicity, there were 118 (55.1%) Caucasians, 12 (5.6%) Black/African Americans, 11 (5.1%) Hispanics, five (2.3%) Asian/Asian Americans, and three (1.4%) Native Americans. The majority of participants reported personally knowing someone who is estranged from a parent (known estranged *n* = 127, 84.1%; did not know anyone estranged, *n* = 24, 15.9%). Participants were also asked if they themselves had ever completely ceased contact from a parent; 79 participants (52.3%) reported that they had, and 72 participants (47.7%) had not.

### 2.2. Procedures and Instrumentation

Upon receiving approval from the University’s Institutional Review Board, recruitment advertisements posted on Amazon Mechanical Turk (MTurk) stated a requirement of being at least 18 years of age to participate. Those who elected to participate clicked a link to an online survey that took about 20 minutes to complete and contained the following measures and aforementioned basic demographic information. Participants were paid 50 cents for completing the survey.

#### 2.2.1. Stereotype Content

Participants were asked to write the “words and phrases that you have heard others say about people who cease contact with a parent,” including “those words or phrases that come to mind, even if you disagree or believe them to be inaccurate.” This procedure follows those of several communication scholars interested in determining the stereotypes publicly held of targeted groups [26,41,42].

#### 2.2.2. Attitudes toward Estranged Adult Children

Positive and negative attitudes toward estranged adult children were measured using Alwin’s [43] Feelings Thermometer. This measure provides research participants the opportunity to report their opinions on different groups of individuals on a familiar tool. This one-item measure captures responses on a 0 to 10 point Likert-type scale ranging from *cold and unfavorable* (0) to *warm and favorable* (10), with the following responses found within our sample: *M* = 5.46, *SD* = 2.63.

#### 2.2.3. Warmth and Competence of People Estranged from a Parent

Perceptions of warmth and competence were measured using a two-dimensional scale intended to rate how participants perceive the target group on two items for each construct: *competent* and *capable* (competence)*,* and *warm* and *friendly* (warmth)*,* respectively [44]. Responses used a 5-point Likert scale ranging from *not at all* (1) to *extremely* (5). Both warmth (*M* = 2.61, *SD* = 1.11, ICC = 0.86) and competence (*M* = 3.31, *SD* = 0.94, ICC = 0.81) measures were reliable.

#### 2.2.4. Personal History of Estrangement

Personal history of estrangement was assessed through two questions: “Are you estranged from a parent?” and “Do you know someone who is estranged from a parent?”

#### 2.2.5. Accommodative Strategies

In order to address RQ3, participants were asked four open-ended CAT-based questions generated to assess the strategies that people think they would employ when communicating with someone they knew to be estranged from a parent. While accommodation is often studied through observations or through a survey asking participants to rate the degree to which they engage in specific behaviors shown previously to decrease or increase social distance [35], there are no preceding studies of strategies directed toward our study’s context of estranged adult children, and so this method serves as a basis for later works on specific accommodative and nonaccommodative behaviors. The language is based off of the theory’s description of accommodations as adjustments or shifts in verbal (e.g., topics, phrases) and nonverbal behaviors (e.g., vocalics, proxemics) [35]: “What, if any changes would you make about the things you say when communicating with individuals who are estranged from a parent (e.g., topics, phrases)?” “What, if anything, would you avoid saying when communicating with them?” “What, if any, changes would you make about how you talk to them and your actions (e.g., gestures, distance, volume)?” and “What, if any, specific things would you avoid doing in this interaction?”

## 3. Data Analysis and Results

In solidifying the stereotypes that Americans hold of those estranged from their parents (RQ1), hypothesis one asserted that these would be more negative than positive in nature. There were 149 participants who provided a response to this question. To analyze our open-ended data, we mirrored quantitative processes of previous stereotype-uncovering studies [26,41,42]. Similar to a content analysis in which open-ended data is systematically quantified, this process of data reduction focuses on systematically reducing data as opposed to interpreting its meanings [45]. After independently reading each participant’s list of stereotypes (*n* = 788), our research team proceeded with the following steps.

First, all but the first author collaboratively placed all of the responses into three groups based on the *object* of the stereotype: stereotypes of adult children in estranged relationships (*n* = 600), stereotypes about the parents in estranged relationships (*n* = 187), and 15 additional “personal disclosures.” Straying slightly from the steps taken in aforementioned stereotype studies, we separated these “personal disclosures” from further analysis due to participants’ diversion from the prompt. Example responses among the 15 are “I have done this to my Mother and my daughter has done this to me” and “I’m personally estranged from my daughter.” Due to the nature of the prompt (i.e., asking about the adult child), the researchers categorized all other responses as stereotypes about the adult child. 

Second, identical and synonymous words or phrases were grouped together separately within either the children or parents stereotype group. In the third phase, similar words—as agreed upon by the research team—and phrases were matched together within each category to create 22 stereotypes of adult children who are estranged and 11 stereotypes of parents of estranged children. After these three phases were presented to the first author, she and the second author revisited the codes in their entirety, and decided to split the first child-focused category into two categories, alter several labels (e.g., “healing” to “moving on”) and move a few individual responses based on these modified category conceptualizations and labels. These modifications to the original category system were all reviewed and approved by the full research team.

The following stereotypes had the highest frequency of unique identifiers (*n* > 21 as this value is 10% of our sample size, see [26]. A binomial distribution determined that 50 responses was a minimum for inclusion in our results [42], but because we had the two separate groups (i.e., parent as object, child as object) we deemed all categories worthy of inclusion. All stereotype categories, frequencies of occurrences, and examples are listed in Table 1.

Among all generated stereotypes (see Table 1), the most common stereotype of adult children who are estranged was *childish/immature* (*n* = 118), and included the terms stubborn, selfish, and brat. Although this first category focused on immaturity, the second most common stereotype was *ungrateful* (*n* = 89), strongly placing children at fault. It included these terms: arrogant, wrong, lacking morals, and other descriptions of intense ingratitude. The third category, *independent and strong* (*n* = 66) included being brave, empowered, and aware that not all family members are positive influences. The fourth most common, *something bad happened* (*n* = 64), included situational factors such as one having legitimate reasons for estranging, being traumatized by childhood, and it involved an assumption that something “deeply unsettling” must have happened. The fifth most frequent, *cold/cruel* (*n* = 61) noted manipulations and/or a lack of warmth toward the parent and/or toward society. *It’s their fault/black sheep* (*n* = 45) addressed the child’s receipt of negative judgment. This category included participants referencing estranged adult children as negative *or* stating that they received negative judgments from others. *Abused* (*n* = 25) included claims of the estranger receiving physical, sexual, or mental abuse. To clarify, the category of *moving on* stereotypes referenced action, whereas *better without parent* stereotypes positively framed the child’s leaving.

All of the parent-targeted stereotypes were those that explicitly focused on the parents’ actions or dispositions, despite no prompt to do so. Again, the following stereotypes had the most frequency of unique identifiers (*n* > 21), though all are listed in Table 2. The most common stereotypes of parents who have estranged children were that they are *abusive* (*n* = 37) and that they *mistreat* (*n* = 34) their children. Within the *abusive* category, *physical abuse* (*n* = 5), *emotional abuse* (*n* = 4), and *mental abuse* (*n* = 4) were all included. *Mistreatment* included stereotypes of abandonment from parents, being wronged by parents, and living in toxic parental situations, but these included no reference to the violence and/or fear of the first category. The third most common stereotype listed the parent as *absent* or *disinterested* in their child (*n* = 29), often citing the absence of behaviors or an overall apathy toward children or childrearing. The fourth most common stereotype was *disapproving*/*difference in beliefs* (*n* = 26) of various kinds and in a general sense. The fifth most common stereotype was *alcohol/drug abuse* (*n* = 22). This category was of stereotypes surrounding the parent’s bad behavior; these included addictions, coinciding illegal activity, or jail time.

All parent-focused stereotype categories, frequencies of occurrences, and examples are listed in Table 2.

Although we note that stereotypes—in and of themselves—are not always *either* negative or positive (e.g., the stereotype that one is good with money can be negatively valenced as “cheap” or positively valenced as “thrifty”), the majority of the responses—which included the child *and* the parent—reflected blatant negative traits, behaviors, or circumstances. Exceptions to these were two categories within the child data: *independent and strong* (*n* = 66) and *moving on* (*n* = 13) whose combined frequencies (*n* = 79) were far outnumbered by the 521 within this group. All of the parent-object stereotypes were negative in nature. Therefore, hypothesis one was supported in that negative stereotypes surpass positive stereotypes of estrangement.

The second research question inquired as to the warmth and competence perceptions of individuals who are estranged. These are typically assessed in relation to warmth and competence held about several unique social groups, with the researchers then using a cluster analysis to determine similarities between and differences across different targeted groups [44] showed how much more warm and/or competent some groups are perceived as being over other groups), but our assessment of *only* estranged individuals called upon us to determine if the mean scores for warmth and competence were statistically different from each other. A paired samples *t*-test showed warmth (*M* = 2.61, *SD* = 1.11) to be significantly lesser than competence (*M* = 3.31, *SD* = .94), *t* (161) = −8.75, *p* < 0.001, *d* = 0.66. These scores hovered around the mean, but their moderate scores’ slight difference from each other suggests that estranged children are seen as slightly more competent than they are warm, and this served as our answer to the second research question.

Hypothesis two asserted that contact with an estranged individual improves attitudes. Results of this independent-samples *t*-test of those self-identifying estranged versus other non-identifying participants yielded significance regarding perceived warmth, *t* (145) = 2.52, *p* = 0.01, *d* = 0.40. This indicates that estrangers and estrangees, compared to the un-estranged, perceived people who are estranged as higher in warmth (*M* = 2.85, *SD* = 1.22) in comparison to the perceptions held by people who are not personally estranged (*M* = 2.40, *SD* = 0.94). There were also significant differences for perceptions of competence *t* (148) = 1.97, *p* = 0.05, *d* = 0.32. This finding indicates that people who are estranged perceive those in this category as higher in competence (*M* = 3.47, *SD* = 0.99) than what is thought by people who are not personally estranged (*M* = 3.17, *SD* = 0.90). Furthermore, there were significant differences in attitudes: *t* (148) = 4.76, *p* < 0.001, *d* = 0.78. Participants who reported being estranged rated estranged children as warmer and more favorable on the Feeling Thermometer (*M* = 6.44 on a 10 point scale, *SD* = 2.29) as compared to other participants’ reported attitudes (*M* = 4.53, *SD* = 2.62).

Our second difference test, comparing scores of those who knew an estranged adult child with scores of those who did not report knowing an estranged child, showed no significant differences across any of the assessed variables. These findings were as follows: warmth, *t* (149) = 0.341, *p* = 0.74; know estranged *M* = 2.65, *SD* = 1.11, do not know estranged *M* =2.56, *SD* = 1.15); competence *t* (148) = 1.78, *p* = 0.09, know estranged *M* = 3.39, *SD* = 0.94, do not know estranged (*M* = 3.00, *SD* = 1.00); and favorable attitudes *t* (148) = 1.66, *p* = 0.71; know estranged, *M* = 5.67, *SD* = 2.57 and do not know estranged (*M* = 4.71, *SD* = 2.84).

A third difference test placed the self-estranged and those who knew an estranged person together. A Welch’s independent samples *t*-test showed no significant mean difference for perceived warmth between these two groups, *t* (23) = 1.30, *p* = 0.21, *d* = 0.32. Participants with a personal history with estrangement (either self or known other) did not perceive estranged individuals as warmer (*M* = 2.68, *SD* = 1.11) compared to participants with no personal history with estrangement (*M* = 2.32, *SD* = 1.16). Results showed significant differences between participants in regard to competence, *t* (23) = 1.30, *p* = 0.03, *d* = 0.55. Participants with a personal history with estrangement perceived estranged individuals as more competent (*M* = 3.40, *SD* = 0.94) than what was perceived by participants with no personal history with estrangement (*M* = 2.87, *SD* = 0.98). Another *t*-test showed significant differences between the two groups, *t* (24) = 2.39, *p* = 0.02, *d* = 0.58 in terms of their attitudes toward estranged individuals. Participants with a personal history with estrangement (*M* = 5.71, *SD* = 2.59) had more positive attitudes toward estranged individuals than that held by participants with no personal history with estrangement (*M* = 4.21, *SD* = 2.55). To conclude, hypothesis two was not supported as differences only emerged for those who were estranged themselves, not for those who merely knew someone who was estranged.

RQ3 asked about the ways Americans would accommodate their speech when in contact with an estranged adult child. The research team compiled 161 discrete responses from *n* = 145 participants who completed this section of questions. Having applied the theory within previous studies, the first author directed the other authors in their understanding and application of the theory’s axioms and definitions. The researchers applied this knowledge in discussing how consistently used CAT terminology of *accommodation*, *overaccommodation*, *underaccommodation*, *reluctant accommodation*, and *avoidant communication* [35] should be employed. We collectively decided that the responses were in accordance with the first three of these CAT phenomena, but that placement of responses in the latter would require inference. If a participant set out to avoid mentioning anything about family or their parents, their response would be placed within “overaccommodative.” This is because such communication is ‘overdoing’ the appropriate accommodation of being mindful of and/or sensitive to this topic. If participants reported being careful as to how to bring up the estranged adult child’s parents, and expressed a sense of support and a consideration of the person who is estranged, it was placed within “accommodative communication.” If the participants stated a desire to make no change to their communication whatsoever, it was placed within “underaccommodative” communication for its complete lack of intended convergence, though we note that the intention *might* be to communicate based on evidenced individual needs within the interaction, we cannot infer this from this data. We independently coded the first 18 responses, resulting in perfect reliability, thus establishing the coding scheme as valid. The data was then split and independently coded among all but the first author who later reviewed their coding scheme agreed with their coding scheme.

Results regarding (non)accommodation intentions toward estranged adult children are as follows: nonaccommodative (*n* = 96) responses outnumber accommodative (*n* = 65). Within the umbrella category of accommodative communication, all responses are purely accommodative (*n* = 65). As noted above, there were no reluctantly accommodative responses. Within the umbrella response of nonaccommodation, the most common responses involved over-accommodation (*n* = 61). The second-largest subgroup of participants mentioned topics placed under the category of underaccommodation (*n* = 30). A portion of the underaccommodative responses was notably insensitive to the topic, citing intended statements such as “get over it.” A few participants (*n* = 5) included avoidant communication in their responses mentioning that they would “feel physical and emotional distance,” feel “very tense,” and “be very short and cold” when speaking to an estranged individual. Although the last of these speaks slightly to the reluctant communication addressed within the theory, there was not substantial evidence within these responses to justify its inclusion as a subcategory.

## 4. Discussion

Alongside scholarship highlighting the relational properties of estranged family dynamics [6,10], this study’s intergroup/social identity approach demonstrated the following: (1) estranged adult children *and* their parents are deemed in primarily negative and some positive ways, (2) compared to their presumed competence, the presumed warmth of estranged children reflects a negative social judgment, (3) there is insufficient evidence of the contact hypothesis in this context, but evidence that self-identifying estranged adult children have more favorable attitudes, and (4) intended behaviors toward estranged adult children are more non-accommodative than accommodative. Combined, these findings amplify understanding of the communication surrounding estranged family relationships and lend themselves to further assessments that address both the negative cognitions and their potential pathways to negative versus positive communication exchanges.

### 4.1. Assigned Blame to the Parent and the Child

Much as we admittedly commit in our introduction to the topic, many in our sample assumed that the estranger is the child, the estrangee, the parent, but this is not always the case [46]. This may be because of the way we presented our questions, an obvious consideration for our results and decision-making in future assessments. Still, within the lines of logic that the child is “more” active in the distancing, we see several trends of blaming. Estranged children, though often victims themselves to painful family circumstances [6,10], are subject to others’ judgment of them as childish, ungrateful, and cruel. These sentiments stand in complete juxtaposition to estrangers’ accounts of “having to” distance themselves, and often suffering great turmoil alongside this difficult decision [7]. Among this sample’s negative stereotypes and attitudes of estranged children, perhaps the most troublesome is the perception that people who are estranged lack warmth. The concern is not in the value itself—with a mean just below the scale midpoint—but in knowing through previous research that groups who are seen in this ways are viewed with contempt [47,48]. So while estranged children’s deemed high(er) competence is a potentially positive thing, societal reactions to them would likely be enhanced if they were known as not just skilled, but also *good* people.

The adult child is not solely blamed, however, as many noted the adult children to be the victims, with many other stereotypes noting the parents’ shortcomings, even in the absence of a specific prompt to write about the parent’s role. Acknowledgement of the family system (or at least one dyad within the system) was apparent, as was the notion that the parents likely hold more power/responsibility than the child, as many participants talked about parents (rather than children) as the objects of their first listed stereotype or as their only listed stereotypes. Stereotypes also reveal that the public misperceives estrangement as an “event” or series of events (“*something bad happened*”), failing to see it as a complex process that occurs over time, often encompassing decades that may involve oscillation between various stages of connection [7].

Unfortunately, amidst noted causes of estrangement, participants failed to directly address the communication problems likely underlying and used to enact the estrangement [10,15,49]. Expressiveness and structural traditionalism are often at play amidst the addiction, illegal activity, and other problems in these family systems [50], and future goals might be to increase understanding and support. While more research leads to the normalization and validation of this family form, Scharp and colleagues point out scholars’ assumptions about the non-obligatory, enduring nature of family [51]. They would probably agree, as we do, that the literature still broadly portrays it as a violation from what families *should* be and what they “naturally” are [5]. Scholars might also address gender differences that, surprising given aforementioned gendered expectations, were less prominent among our participants responses, though they may or may not still be presumed (and just unacknowledged).

### 4.2. Pathways to (Positive) Communication with People who Are Estranged

A plethora of previous research suggests that a primary pathway to more pro-social interactions is the simple interaction between outgroup members. Allport’s contact hypothesis and its more complex successors (e.g., common ingroup identity model, [52]) reveal the benefits of contact, particularly if it is positive. Our sample yielded no significant differences in prejudices based on participants’ knowledge of an estranged individual, but it did reveal that those with some direct involvement with estrangement themselves had more positive perception of those who are estranged. Specifically, compared to those who did not know an estranged adult child, participants directly involved in estrangement perceived estranged adult children as more competent, and as warmer, and they also held more favorable attitudes of (other) estranged adult children. As estrangement is a very painful process, and often includes feelings of guilt and shame, these findings offer some hope. It is important to emphasize, however, that estrangers and estrangees are not reporting positive attitudes about the circumstance of estrangement, only about those *living* with estrangement. Perhaps intergroup contact does improve these attitudes, but because so few individuals reported knowing an estranged child, we did not have the statistical power to demonstrate this. If estrangement were less taboo, perhaps more estranged adult children would disclose their estrangement rather than holding back for fear or rejection, and thus future studies might then demonstrate the positive benefits of this specific type of intergroup contact.

When intergroup contact did occur among those in our study, it may have been perceived as negative. People experiencing estrangement tend to disclose to a select few [53], and so those who are talking with a known estranged child (or parent, sibling, etc.) may have negative feelings associated with this task, including sadness from learning more about the process of estrangement that the discloser is experiencing, and may have been experiencing much or all of their lives. Given that the process of estrangement leads some people to develop insecurities and habits that many self-report as impacting their later-life relationships [11], it is also possible that some respondents are *in* that later-life relationship, or are observing it. An obvious limitation, then, is that we know less about the nature of the participants’ direct and indirect experiences with estrangement. Knowing the relationship type, for instance, would be useful given that prejudice-reducing power of close interpersonal relationships such as friendships and romantic relationships [54]. It would also be useful to know the relationship length, level of closeness, and specific affect felt for the individual. Among these, degrees of positive “versus” negative contact may be the best next step for researchers.

While negative contact can still improve attitudes [55], those with favorable experiences report optimum results in terms of lower intergroup bias in affective reactions and overall attitudinal favorability [56]. While difference tests cannot confirm the causal mechanisms presumed by the contact hypothesis, they are in accordance with it. The idea that intergroup contact of this nature would be perceived negatively is apparent in the responses that participants would employ non-accommodative strategies of attacking or avoiding the estranged adult child.

Accommodating and nonaccommodative strategies yield some hope, also some concerns, when considering the interactions likely occur with people who are disconnected from their family members. While some among those we sampled are sensitive and responsive in their accounts of how they would intend to communicate with someone known to be estranged from their family member, they show little evidence that they would be excited to have these interactions. Because child-targeted stereotypes were often condemning, it makes sense the majority of participants reported using more divergent, as opposed to convergent, strategies. The participants’ desires to avoid discussing topics that might be upsetting to the estranged individual, choosing to not mention family at all, and completely refraining from conversations are similar to those of distancing, excluding, and rejecting that have been shown of other social groups that are similarly perceived as lower in warmth than in competence [47,48]. This matters, as underaccommodative behaviors are dissatisfying, and also oppositional to those circumstances best suited for building a meaningful relationship with an estranged individual—one of the best pathways to improved intergroup dynamics (optimum intergroup contact [57]). One must also consider that one’s identity as an estranged parent or adult child is most likely hidden during initial (and perhaps long-standing, should it be kept a secret by the person who is estranged) interactions with acquaintances, which might also alter the dynamic of intergroup exchanges. These negative sentiments might also coincide with intergroup anxiety, and thus can be strong deterrents in reducing prejudice [58].

As one of our reviewers pointed out, there may be some accommodative functionality to people’s aversion to talking about estrangement (CAT positions accommodation in the eye of the beholder, and so there can be exceptions to the “convergent strategies lead to accommodation” trend that is commonly observed). The existent cross-contextual accommodation-based literature gives little attention to this possibility. Unlike social identities of culture, race, age, gender, and countless others, those experiencing estrangement are working—some struggling—to make sense of the situations for themselves [51], some even hiding the information from close others and their medical professionals [11]. In addition to the stigma experienced by children and parents who are estranged, the family communication that surrounds the estrangement (e.g., abuse, betrayal, exclusion) is likely painful, even further stigmatizing, to discuss. Unlike asking someone about their heritage or faith, for instance, asking someone about their estrangement might be met with mixed or highly negative sentiments due to the content they are prompted to disclose. As Agllias [7] attests, even when people say that they are satisfied with their discontinuation of contact, they often contradict themselves by stating their pain, implying their feelings of guilt, or crying and/or yelling in order to release their negative emotions. Also, depending on where someone’s circumstances reside on what Scharp and colleagues label the *estrangement continuum* [6], they may not even self-identify *as* being estranged from their family member. What might be most empowering, then, is to focus on the hope and resilience of those who are estranged [11], as they learn to decide when and how to “come out to” their loved ones. Doing so will certainly be easier when cultural expectations change, and such is the basis of our intergroup approach to this topic.

In short, while there is reason to believe that communication with and about estranged family members contains sympathetic sentiments, negative judgment and reluctance present concern for those interested in enhancing the lives of those who are experiencing estrangement. Certainly, in accordance with social identity theory [21], there is hope in that those who *are* estranged see their own group in a (more) positive and accurate light, as opposed to a more self-deprecating one. Given the sadness and isolation felt by those living these delicate family dynamics, we look forward to future scholarship demonstrating the ways their communication can be enhanced. In doing so, we encourage scholars to also be mindful of our study’s generalizability—reducing limitations of cross-sectional design, failure to directly assess emotions, and employment of only MTurk participators who own a computer and selected, rather than were randomly assigned to, participate in this study.

## 5. Conclusions

This study provides a new context through which we see society placing judgment about an individual based on his/her family structure. While there are many stereotypes of estranged adult children, and negative attitudes and general perceptions that they are somewhat lacking in warmth, there is also evidence that the public deems estrangement as something that is undeserving of these negative judgments (i.e., the moderate competence perceived of estranged adult children, stereotypes with emphasis on their *parent’s* shortcomings). Because stereotypes are shown to fuel behaviors such as those suggested in our participants’ accommodative and nonaccommodative intended behaviors toward those who are estranged, we challenge researchers to move beyond cross-sectional data to ask a broader public about their agreement with and negative/positive evaluations of these demonstrated stereotypes, to directly experiment with stereotype primes and communicative outcomes, and to employ emotions as they act as a powerful mediator between stereotypes and behavioral outcomes [31]. In doing so, we might see how gendered norms of accommodation (i.e., women converging more [59]) coincide with gendered expectations of estrangement. As good communication is a primary means of reducing social psychological distance between individuals and groups, we encourage studies and applications dedicated to reducing anxiety and ultimately improving empathy and support for people managing these difficult family communication processes.

## Figures and Tables

**Table 1:** Stereotypes of adult child estranged from a parent (*n* = 149).

Stereotype Category	Frequency (*n*)	Examples
Childish/Immature	118	“Selfish,” “Stubborn,” “Rebellious”
Ungrateful	89	“Ungrateful beasts,” “Disrespectful,” “Unappreciative of parents”
Independent and Strong	66	“Stands up for themselves,” “Resilient,” “Determined to make their own destiny”
Something Bad Happened	64	“Toxic relationships,” “Traumatic interactions,” “Have legitimate reasons”
Cold/Cruel	61	“Uncaring,” “Angry,” “Vindictive”
It’s Their Fault/Black Sheep	45	“Looked down upon,” “I think they are messing up their life,” “The ones to blame”
Abused	25	“Childhood abuse,” “Physical abuse,” “Mental abuse”
Better without Parent	20	“As if a weight has been lifted,” “Free”
Complicated/Puzzling	18	“Hard to understand,” “Issues,” “Families are very complicated”
Mental Problems	17	“Depression,” “Broken,” “Crazy”
Out of Options	16	“Met their breaking point,” “At their wit’s end, “They had no choice”
Dislike Authority	14	“Defiant,” “Sick of having someone else telling them what to do”
Moving On	13	“Setting boundaries with people whom might have harmed them,” “Protecting themselves”
Overreactive	10	“Overreacting to a situation,” “Being over dramatic,” “Overly sensitive”
Alcohol/Drug Abuse	9	“Alcohol abuse,” “Drug abuse,” “Addiction problems”
Sinful	8	“Fake Christian,” “Devil’s spawn”
Pitiful	7	“I feel sorry for them,” “missing out,” “They are a victim”

**Table 2:** Stereotypes of a parent estranged from adult children (*n* =149).

Stereotype Category	Frequency (*n*)	Examples
Abusive	37	“Parent abusive,” “Emotionally abusive,” “Don’t feel safe with that parent”
Mistreatment	34	“Parents treated him horrible,” “Saying mean things to them.”
Absent/Disinterested	29	“Neglectful parents,” “Unsupportive,” “Parent lacks compassion”
Disapproving/Belief Differences	26	“Parent disagrees with child life choices,” “You’re no son/daughter of mine,” “Parent was being unreasonable”
Alcohol/Drug Abuse	22	“Drug problem,” “Alcohol problems,” “Parent is involved in dangerous activity”
Financial Mistreatment	8	“Parent kept asking for money,” “Debt issues,” “Parents stole from them.”
Controlling/Interfering	7	“Tries to run my life,” “Overbearing”
Favoritism	7	“Prefers other siblings over them,” “Step-parents,” “The parent always chose a partner over them.”
Selfish	6	“Only cares about herself,” “Self-centered”
Abandonment	6	“Their parents disowned them”
Mental Problems	5	“Crazy,” “Personality disorders”
